# Lung Resections for Elderly Patients with Lung Metastases: A Comparative Study of the Postoperative Complications and Overall Survival

**DOI:** 10.3390/curroncol29070357

**Published:** 2022-06-26

**Authors:** Mohamed Hassan, Benjamin Ehle, Bernward Passlick, Konstantinos Grapatsas

**Affiliations:** 1 Department of Thoracic Surgery, Medical Center-University of Freiburg, 79106 Freiburg im Breisgau, Germany; benjamin.ehle@uniklinik-freiburg.de (B.E.); bernward.passlick@uniklinik-freiburg.de (B.P.); konstantinos.grapatsas@uniklinik-freiburg.de (K.G.); 2 Faculty of Medicine, University of Freiburg, 79106 Freiburg im Breisgau, Germany

**Keywords:** metastases, metastasectomy, elderly

## Abstract

Background: Pulmonary metastasectomy (PM) is an established treatment option for selected patients with stage IV solid tumors. The aim of this study was to investigate the feasibility of and survival rate in PM for elderly patients. Methods: We retrospectively analyzed all of the patients who underwent PM with curative intention at our institution. The patients were categorized into two groups: the elderly group (≥70 years old) and the non-elderly group (<70 years old). Results: The elderly group consisted of 222 patients versus 538 patients in the non-elderly group. The median number of resected metastases was 2 ± 3 in the elderly group and 4 ± 5 in the non-elderly group (*p* < 0.01). No difference in the rate of postoperative complications was observed between the two groups (*p* = 0.3). The median length of hospital stay in each group was comparable (10 ± 5 vs. 10 ± 4.3 days, *p* = 0.3). The 5-year survival rate was 67% in the elderly group and 78% in the non-elderly group (*p* = 0.117). In the univariate analysis, COPD was associated with poor survival in the elderly group (*p* = 0.002). Conclusion: The resection of pulmonary metastases in elderly patients is safe, is not associated with increased risks of postoperative complication, and the survival benefit is not reduced in selected patients.

## 1. Introduction

The lung is one of the most common sites for tumor metastases. Today, pulmonary metastasectomy (PM) is a safe and well-established treatment that can prolong survival rates in selected patients. General accepted oncological criteria for performing PM include the oncological control of the primary tumor, the lack of extrathoracic metastasis and the resectability of the lung metastases, and an adequate pulmonary reserve [1]. It can be assumed that patient’s age plays an important role in the referral to the thoracic surgeon for PM, and as a result, many elderly patients could be undertreated and excluded from receiving potential curative surgery [2,3,4].

Colorectal cancer is the commonest tumor entity for which PM is indicated. However, prolonged survival after PM has been shown in retrospective studies for germ cell tumor, melanoma, sarcoma, thyroid, and renal cancer [1]. In the existing literature, there is a plethora of information regarding prognostic factors after PM. However, the issue of PM and its results in the elderly population have not been thoroughly examined [5,6]. The aim of this study was to investigate the morbidity and mortality rates associated with PM in the elderly population and to identify risk factors for morbidity and survival in this population.

## 2. Materials and Methods

In this retrospective study, we included all the patients with pulmonary metastases, from a variety of primary tumors, who underwent PM with curative intent at our institution. Tumor entities included melanoma, germ cell tumor, renal cell carcinoma, colorectal cancer, thyroid cancer, ovarian, cervical, and endometrial cancer, breast cancer, head and neck cancer, osteosarcoma, soft-tissue sarcoma, as well as other malignancies (for example, gastric cancer or bladder cancer). Demographic, clinical, and follow-up data were extracted from electronic medical records between January 2000 and December 2020. Included patients were operated in curative intent according to general accepted criteria. Patients selected for PM with curative intent should have a good general condition. All the lung metastases should be surgically resectable. The exclusion criteria were presence of extrathoracic metastases at the time of surgery, pleural carcinomatosis, carcinomatous lymphangiosis, and patients with end stage COPD in GOLD stage IV.

The patient population was divided into two groups: elderly patients and non-elderly patients. Elderly patients were defined as the patients who were aged 70 years or more. The non-elderly group included patients who were aged < 70 years.

Disease-free interval (DFI) was defined as the period between treatment of the primary tumor and PM. Overall survival (OS) was calculated between the first PM and the death of the patient from any cause or the last date on which the patient was known to be alive. Mortality was subdivided into in-hospital mortality and 30-day mortality. Follow-up was performed up to the 73rd month postoperatively.

Video-assisted thoracoscopic resections (VATS) or open lung resections with thoracotomy, as well as the extent of the resection, were decided upon according to the number, size, and location of metastases. PM was conducted with wedge resections, segmentectomies, lobectomies, or pneumonectomies. PM of more than 4 lung metastases per patient was defined as the resection of a high number of metastases.

### 2.1. Definition of Complications

Morbidity or complications were defined as the appearance of new disease during the first 30 days after surgery which required specific treatment and/or implied an increase in the length of hospital stay. Postoperative pneumonia was diagnosed according to the European Perioperative Clinical Outcome (EPCO) criteria [7], which include new pulmonary infiltrations with associated leukocytosis, fever, new purulent sputum, required antibiotic therapy, and increased oxygen demand via face mask. Air leaks were considered persistent if they were present for more than 6 days. The distinction between minor and major complications was made according to the Clavien–Dindo classification. Clavien–Dindo grade I–II complications were defined as minor complications. In this case, treatment only consisted of medication or minor interventions without general anesthesia. Clavien–Dindo grade ≥III complications were defined as major complications and included surgical, radiological, and endoscopic intervention under general anesthesia, intensive care, and life support, or the presence of single- or multiorgan dysfunction [8].

### 2.2. Definition of Comorbidities

Comorbidities such as diabetes mellitus, cardiac comorbidities, and COPD were examined. Cardiac comorbidities included reduced left ventricular function, arrythmias, coronary heart disease, valvular heart disease, history of previous cardiac operations, and history of myocardial infarction. COPD included patients with COPD stage I–III according to GOLD criteria [9]. No patient with COPD GOLD IV was identified, and for this reason, no patient was included in this category.

### 2.3. Data Collection and Statistical Analysis

The study was conducted in accordance with the Declaration of Helsinki (as revised in 2013). The study was approved by our local ethics committee and registered in the German Registry for Clinical Trials (DRKS-ID: DRKS00021251). The need for informed consent was waived due to the retrospective nature of the study. Morbidity and mortality rates were analyzed using cross tables, the χ2 test, and Fisher’s exact test. Survival curves were estimated using the Kaplan–Meier method. The log-rank test was used to calculate survival differences between the groups. A stepwise backward multivariate Cox proportional hazard model was used to evaluate prognosticators on survival benefits and possible risk factors for postoperative morbidity. All the analyses were performed using the SPSS 22.0 software (SPSS Inc., Chicago, IL, USA). The results were considered statistically significant if the *p* value was less than 0.05.

## 3. Results

### 3.1. Age Characteristics of the Study Population

The patients were subdivided into two groups according to their age. In total, 222 patients (29.2% of the study population) were included in the elderly group (≥70 years old), and 538 patients (70.8%) in the non-elderly group.

### 3.2. Preoperative Comorbidities in the Study Population

Elderly patients undergoing PM were preoperatively diagnosed with more cardiac comorbidities (*n*: 42 patients, 18.9% in the elderly population vs. *n*: 33, 6.1%, *p* < 0.001). In addition, diabetes mellitus was more often diagnosed in elderly patients (*n*: 24 patients, 10.8% vs. *n*: 29 patients, 5.4% of non-elderly patients, *p*: 0.008). However, the rate of COPD was similar in both groups (*n*: 17 elderly patients, 7.7% vs. *n*: 57 young patients, 10.6%, *p*: 0.2). Data concerning preoperative comorbidities are summarized in Table 1.

### 3.3. Primary tumor

The most common primary tumor in elderly patients undergoing PM was colorectal cancer (*n*: 112, 50.7%), followed by renal cell cancer (*n*: 27, 12.2%) and soft-tissue sarcoma (*n*: 13, 5.9%). The histological types of the primary tumors in both age groups are described in Table 2.

### 3.4. Surgical Characteristics

In total, 989 lung resections were performed as first PMs in 760 patients. Non-elderly patients were more often bilaterally operated upon than elderly patients (*n*: 205, 39.3% vs. *n*: 51 patients, 14.2%, *p* < 0.001). In addition, fewer sequential thoracotomies were performed in the elderly group (*n*: 41 patients, 18.5% vs. *n*: 166, 30.9%, *p* < 0.01). Moreover, more minimally invasive video-assisted thoracic surgery (VATS) was performed in the elderly (*n*: 77 patients, 34.7% vs. *n*: 115, 21.4%, *p* < 0.001). The type of lung resections performed was similar in both groups. The surgical characteristics of the patients are summarized in Table 3.

### 3.5. Resected Lung Metastases

Fewer lung metastases were resected in elderly patients in comparison with younger ones (2.3 ± 2.6 lung metastases vs. 4.1 ± 5.4 in younger patients, *p* < 0.01). In addition, more single-lung metastases were resected in elderly patients (*n*: 118, 53.2% vs. *n*: 218, 40.5%, *p*: 0.001). In both groups, similar incidence rates of intrapulmonary lymph node metastases (*n*: 6, 2.7% in elderly vs. *n*: 28, 5.2%, *p*: 0.1) and mediastinal lymph node metastases (*n*: 15 elderly patients, 6.8% vs. *n*: 49, 9.1%, *p*: 0.2) were detected. Fewer patients in the elderly group underwent an incomplete resection (*n*: 8, 3.6% vs. *n*: 39, 7.3%, *p*: 0.04). Furthermore, more repeated resections were performed in non-elderly patients. The data are summarized in Table 3.

### 3.6. Postoperative Morbidity and Hospital Stay

The postoperative morbidity rate was similar in both age groups (*n*: 44, 19.8% vs. *n*: 125, 23.2%, *p*: 0.3). In both groups, minor (*n*: 33, 14.4% vs. *n*: 99, 18.2%, *p*: 0.2) and major complication rates (*n*: 11, 5% vs. *n*: 26, 4.8%, *p*: 0.2) were comparable. The rates for pneumonia (*p*: 0.8), postoperative atrial fibrillation (*p*: 0.8), intestinal obstruction (*p*: 0.04), surgical revision (*p*: 0.7), postoperative lung embolism (*p*: 0.5), wound-healing disorder (*p*: 0.06), the need for new chest tube placement (*p*: 0.1), prolonged air leak (*p*: 0.9), and postoperative cardiovascular complications (*p*: 0.5) were shown to be similar in both groups. Furthermore, no statistical significance was shown for the length of hospital stay in both groups (10 ± 5 days vs. 9.6 ± 4.3 in non-elderly patients, *p*: 0.3).

### 3.7. Postoperative Mortality

The in-hospital mortality rate was zero in both groups. The 30- and 90-day mortality rates were limited and similar in both groups. The data are summarized in Table 4.

### 3.8. Survival after PM

**Figure 1:**
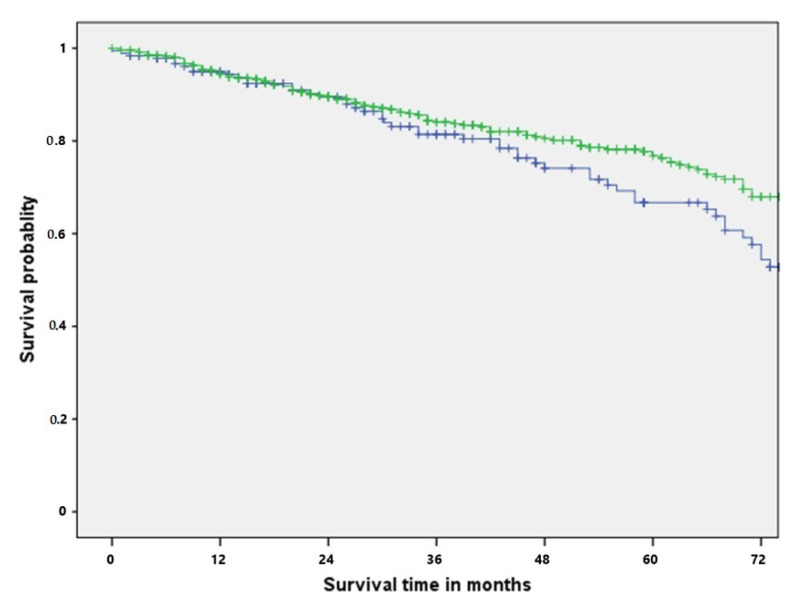
Overall survival rate for elderly (blue) and non-elderly (green) patients undergoing pulmonary metastasectomy.

The DFI as shown to be shortened in the elderly population (45.18 ± 32.70 vs. 73 ± 88.65, *p*: 0.324). However, here, no statistical significance was shown. In the main study, the median overall survival in the population was 40 months (range: 1–223 months). According to the log-rank test, the difference between the survival for the elderly and non-elderly population was not significant (*p*: 0.116). The 3-year-survival rate in the elderly and in the non-elderly population was 83.1% and 86.2%, respectively. The 5-year-survival rate in the elderly group and in the non-elderly group was 66.7% and 77.7%. In the univariate analysis, age > 70 years was not identified as an adverse prognostic factor (HR: 0.779, CI 95%: 0.555–1.068, *p*: 0.117). In the log-rank test, no statistical significance was shown concerning the survival rate between the two groups (*p*: 0.116) (Figure 1).

Five-year-survival for elderly patients with colorectal cancer, renal cell cancer, soft-tissue-sarcoma, and head and neck cancers was 68%, 48.6%, 60%, and 33.3%. Concerning the primary tumor, no statistical significance was found in the log-rank test between the two groups (*p*: 0.510).

### 3.9. Prognostic Factors for Survival and Postoperative Morbidity in the Elderly Population after PM

In the univariate analysis, COPD was identified as an adverse prognostic factor in the elderly population for postoperative morbidity (HR: 9.201, 95% CI: 1.890–44.800, *p*: 0.006) and survival (HR: 7.401, 95% CI: 2.139–25.606, *p*: 0.002). In Table 5 and Table 6, the univariate analysis of the prognostic factors is demonstrated.

## 4. Discussion

Our study’s main finding is that, in elderly patients, PM is a safe method which can offer prolonged survival rates in selected patients. Mortality and complication rate (minor and major) was similar in both age groups. In the elderly, COPD was identified as a negative predictor for postoperative morbidity and an adverse prognostic factor for survival.

Elderly patients with lung metastases are often undertreated because of their age, their increased comorbidities, and their lower life expectancy [10]. In this way, many patients are excluded from receiving potential curative treatment [5,6]. In our study, the safety of the use of PM in the elderly population was depicted. After PM, the postoperative complications were similar in both groups. These results are also similar with the results in the literature. In their study, in which morbidity and mortality was examined after PM for elderly patients with colorectal cancer, Sponholz et al. reported similar results. Here, the rate of minor and major morbidities for the entire study population was 17% and 5.8%, respectively [6]. In addition, in their multicenter study of 32 Spanish hospitals with 532 patients undergoing PM for colorectal cancer, Rodriguez-Fuster et al. reported an incidence rate of 16% of postoperative morbidity [11].

Elderly patients undergoing PM had more preoperative comorbidities such as cardiac comorbidities and diabetes mellitus. Unfortunately, the cause of the cardiac comorbidities could not be identified. Due to the retrospective nature of our study, no information concerning chemotherapy-induced cardiac comorbidities could be provided. However, preoperative comorbidities did not contribute to increased complications in the elderly group. We believe that this is attributed to the fact that the elderly patients in our study were carefully selected, and the treatment for the chronic comorbidities was well adjusted before the surgery. Our findings reflect those of other studies. In the study by Sponholz et al., in the elderly population group, COPD and a Charlson comorbidity index score > 6 were often seen, and in the elderly population, there were trends for heart arrythmia and coronary heart disease. However, in both groups, the postoperative morbidity rate was similar [6]. Furthermore, in the study by Rodriguez-Fuster, despite the comorbidities of the elderly population, age was not identified as a risk factor [11]. An additional possible explanation for these findings is that the elderly patients were protected through the limited resections. In our study, in the elderly population, 92.3% of the lung resections were wedge resections and segmentectomies. Our last hypothesis is also strengthened by results reported after lung resections for non-small cell lung cancer (NSCLC). For example, in the study by Spaggiari et al., elderly patients who underwent anatomical lung resections for NSCLC (71.8% of the study resections) showed significantly higher postoperative morbidity rates (overall postoperative morbidity: 54.2% vs. 41.6%; postoperative respiratory morbidity: 25.7% vs. 8.3%; postoperative cardiac morbidity: 28.5% vs. 11.4%) [12].

Many studies associated with PM have investigated prognostic factors for survival after PM. However, a limited number of studies have investigated predictors for mortality and morbidity after PM in the elderly. To our knowledge, only Barone et al. and Sponholz et al. investigated the outcomes of PM for colorectal cancer in elderly patients [5,6]. Barone et al. reported a postoperative complication rate of 23.8% in 21 patients older than 75 years [5]. Major lung resections and preoperative cardiovascular comorbidity were already identified as risk factors for postoperative morbidity after PM [11]. Heart arrythmia in the elderly was also identified as risk factor for postoperative complications in the study by Sponholz et al. [6]. In our study, in the univariate analysis, COPD was identified as a risk factor for postoperative complications. COPD is already a well-identified risk factor for perioperative morbidity in elderly patients [13]. However, these findings are not in compliance with the results reported by Sponholz et al., in which COPD was not identified as a risk factor [6].

PM was associated with low postoperative mortality (0–2.5%) [1,11,14,15,16,17,18]. For this reason, potential risk factors for mortality could not be assessed. Furthermore, similar results were reported in the studies by Sponholz et al. and Rodriguez-Fuster et al., with zero mortality and 0.37% mortality, respectively [5,11].

In our study, the 5-year-survival rate in the elderly group was reduced in comparison to the non-elderly group (66.7% and 77.7%). However, in this case, no statistical significance was identified (*p*: 0.116). We assume that in this case, elderly patients showed reduced survival due to the natural course of their advanced age. Furthermore, no statistical significance was shown concerning the histological type in the elderly population (*p*: 0.510). PM for colorectal cancer has been identified to prolong survival in selected patients (1, 5, 6). Patients with head and neck tumors and lung metastases are associated with reduced survival rates. Liu et al. reported a 5-year-survival up to 30% [19] We believe that the 5-year-survival of 33% in the elderly group in our study is attributed to a possible selection bias. Furthermore, the lack of statistical significance between the primary tumor subgroups should be interpreted with caution due to the relatively small size of the subgroups.

Prognostic factors for survival could be helpful tools for clinicians to evaluate the treatment of patients. The number of lung metastases, short DFI, and the incomplete resection of lung metastases and lymph node metastases have been identified as adverse prognostic factors for survival [1]. Reports on prognostic factors for overall survival in the elderly undergoing PM are limited [5,6]. We identified COPD as an adverse prognostic factor for survival after PM for elderly patients. We believe that patients with COPD were in reduced general condition and this fact led to a reduced OS rate. Our findings are strengthened by those of Rodriguez-Fuster et al. Here, in the multivariate analysis, preoperative respiratory comorbidity was identified as an independent adverse factor for postoperative morbidity [11]. Concerning the other prognostic factors, we believe that a possible selection bias exists. In this way, patients with a short DFI or lymph node metastases were not referred from the treating oncologists to surgery. A possible selection bias is also verified from the fact that in the elderly population, less lung metastases were resected per patient and fewer repeated operations were performed.

In addition, we believe that lung sparing wedge resections and segmentectomies acted protectively in the elderly population and led to similar morbidity and mortality rates to those of the younger patients, even after the resection of a high number of lung metastases (>4). For this reason, we believe that PM is a safe procedure, even for use in elderly patients. In our opinion, elderly patients should not be routinely excluded from PM with curative intentions. We believe that careful preoperative preparation and selection should take place before the operation, as PM in the elderly can prolong their survival.

In addition, modern cancer treatment shows a promising improve towards precision treatment based on individual genomic profiling [20]. Renaud et al. showed a significant improvement in the survival rate after anatomical segment resections for patients with pulmonary metastatic colorectal cancer with KRAS mutation [21]. Future studies are needed to investigate the benefit of this personalized treatment concept for elderly patients with lung metastases.

### Limitations

Our study is limited by its retrospective nature. Moreover, a selection bias in the choice of elderly patients to undergo PM could exist as elderly patients in reduced general condition or with extensive multiple metastases would not be referred to surgery from the treating oncologists. One additional limitation was the variety of the primary tumors included in the study. For this reason, further investigation of treatments such as tyrosine kinase inhibitors or the influence of gene variations (e.g., K-RAS) could not be performed. Furthermore, due to the retrospective nature of the study and the lack of certain information, further investigation concerning perioperative chemotherapy was not performed. As a result, our study is limited to examining the application of local treatment of pulmonary metastases in the form of surgical resection. For this reason, OS was related only to PM. However, in our study, we examined 760 patients undergoing 989 operations for PM, and a great number of them (*n*: 222 patients) were elderly. We believe that, despite the mentioned limitations, our study represents a good example of everyday clinical practice.

## 5. Conclusions

The use of PM with curative intention in the elderly shows comparable morbidity and mortality rates to those of younger patients and can prolong their survival. For this reason, elderly patients should not be excluded from surgery before careful evaluation. COPD in the elderly is an adverse factor for morbidity and survival.

## Figures and Tables

**Table 1:** Comorbidities in the study population.

Variable	Elderly (*n* Patients, %)	Non-Elderly (*n* Patients, %)	*p* Value
Cardiac comorbidities			<0.001
No	180 (81.1%)	504 (93.9%)	
yes	42 (18.9%)	33 (6.1%)	
COPD I–III			0.2
No	204 (92.3%)	480 (89.4%)	
yes	17 (7.7%)	57 (10.6%)	
Diabetes mellitus			0.008
No	198 (89.2%)	506 (94.6%)	
yes	24 (10.8%)	29 (5.4%)	

Abbreviations: COPD, chronic obstructive pulmonary disease.

**Table 2:** Histological type of the primary tumors.

Histological Type	Elderly (*n* Patients, %)	Non-Elderly (*n* Patients, %)
Melanoma	16 (7.2%)	41 (7.6%)
Germ cell tumor	0	17 (3.2%)
Renal cell carcinoma	27 (12.2%)	57 (10.6%)
Colorectal cancer	112 (50.7%)	244 (45.4%)
Thyroid cancer	6 (2.7%)	12 (2.2%)
Ovarian, cervical, and endometrial cancer	9 (4.1%)	9 (1.7%)
Breast cancer	4 (1.8%)	21 (3.9%)
Head and neck cancer	8 (3.6%)	23 (4.3%)
Osteosarcoma	2 (0.9%)	28 (5.2%)
Soft-tissue sarcoma	13 (5.9%)	48 (8.9%)
Other malignancies	24 (10.9%)	38 (7.1%)

Data are presented as frequencies and percentages.

**Table 3:** Patients’ and surgical characteristics.

Variable *	Elderly (*n* Patients, %)	Non-Elderly (*n* Patients, %)	*p* Value
Patients	222 (29.2%)	538 (70.8%)	-
Male patients	139 (62.6%)	326 (60.6%)	0.604
DFI (months)	45.18 ± 32.70	73 ± 88.65	0.324
Side of pulmonary metastasectomy			
-unilateral	160 (75.8%)	317 (60.7%)	
-bilateral	51 (24.2%)	205 (39.3%)	<0.001
THT/THT	41 (18.5%)	166 (30.9%)	<0.01
VATS	77 (34.7%)	115 (21.4%)	<0.001
Open thoracotomy	145 (65.3%)	423 (78.6%)	<0.001
Wedge resection	170 (76.1%)	412 (76.6%)	0.8
Segmentectomy	36 (16.2%)	97 (18%)	0.5
Lobectomy	15 (6.8%)	25 (4.6%)	0.23
Pneumonectomy	1 (0.5%)	4 (0.7%)	0.65
Number of resected lung metastases	2.3 ± 2.6	4.1 ± 5.4	<0.01
Single metastasis	118 (53.2%)	218 (40.5%)	0.001
N1 lymph node metastasis	6 (2.7%)	28 (5.2%)	0.1
N2 lymph node metastasis	15 (6.8%)	49 (9.1%)	0.2
R1 Status	8 (3.6%)	39 (7.3%)	0.04
Repeated pulmonary metastasectomy	32 (14.4%)	133 (24.7%)	0.002
Second Repeated pulmonary metastasectomy	7 (3.2%)	50 (9.3%)	0.003

* Abbreviations: DFI, disease-free interval; THT/THT, sequential thoracotomy; VATS, video-assisted thoracoscopic surgery. Continuous data are shown as median with range, count data are presented as frequencies and percentages.

**Table 4:** Morbidity and mortality in elderly patients and in patients aged < 70 years.

Variable	Elderly (*n* Patients, %)	Non-Elderly (*n* Patients, %)	*p* Value
Postoperative complication	44 (19.8%)	125 (23.2%)	0.3
Minor complication	33 (14.4%)	99 (18.2%)	0.2
Major complication	11 (5%)	26 (4.8%)	0.2
Pneumonia	12 (5.4%)	27 (5%)	0.8
Atrial fibrillation	3 (1.4%)	8 (1.5%)	0.8
Intestinal obstruction	0 (0%)	6 (1.1%)	0.04
Surgical revision because of complication	7 (3.2%)	15 (2.8%)	0.7
Pulmonary embolism	1 (0.5%)	1 (0.2%)	0.5
Pleural empyema	1 (0.5%)	6 (1.1%)	0.3
Wound-healing disorder	0 (0%)	5 (0.9%)	0.06
Chest tube placement (pneumothorax, pleural effusion)	4 (1.8%)	20 (3.7%)	0.1
Prolonged air leak (>6 days)	2 (0.9%)	5 (0.9%)	0.9
Cardiovascular complication	6 (2.7%)	11 (2.0%)	0.5
Other (e.g., electrolyte disorders)	6 (2.7%)	21 (3.7%)	-
Length of Hospital stay (days)	10 ± 5	9.6 ± 4.3	0.3
In-hospital mortality	0	0	-
30-day mortality	2 (1.1%)	4 (0.8%)	0.7
90-day mortality	8 (4.3%)	10 (2.1%)	0.1
3-year survival	83.1%	86.2%	0.117
5-year survival	66.7%	77.7%	

Continuous data are shown as median with range, count data are presented as frequencies and percentages.

**Table 5:** Univariate analysis of predictors for postoperative morbidity in elderly population.

Variable	Univariate Analysis		
	HR	95% CI	*p* Value
Male patients	1.049	0.387–2.845	0.925
Cardiac comorbidities	1.521	0.494–4.677	0.465
VATS	1.627	0.466–5.678	0.445
COPD I–III	9.201	1.890–44.800	0.006
Diabetes mellitus	0.042	0.01–53.619	0.385
THT/THT	0.961	0.276–3.349	0.95
Lobectomy	0.520	0.148–1.831	0.309

Abbreviations: COPD, chronic obstructive pulmonary disease; VATS, video-assisted thoracoscopic surgery; THT/THT, sequential thoracotomy.

**Table 6:** Univariate analysis of prognostic survival factors in the elderly population.

Variable	Overall Survival		
	Univariate Analysis		
	HR	95% CI	*p* Value
Male patients	1.226	0.651–2.310	0.528
Cardiac comorbidities	1.105	0.489–2.500	0.81
COPD I–III	7.401	2.139–25.606	0.002
Diabetes mellitus	0.831	0.255–2.704	0.759
THT/THT	1.081	0.498–2.346	0.844
VATS	1.650	0.729–3.733	0.230
Lobectomy	0.735	0.261–2.067	0.559
Colorectal cancer	0.960	0.851–1.083	0.506
DFI	1.001	0.999–1.002	0.309
R Status	1.281	0.926–1.774	0.135
N2—lymph node metastases	1.106	0.896–1.365	0.347
Postoperative comorbidity	1.172	0.58–2.653	0.703
Major postoperative complication	0.888	0.169–4.660	0.88
Postoperative cardiovascular complication	0.6	0.09–4.9	0.6
>4 resected lung metastases	0.734	0.261–2.064	0.558

Abbreviations: HR, hazard ratio; 95% CI, 95% confidence interval; COPD, chronic obstructive pulmonary disease; THT/THT, sequential thoracotomy; VATS, video-assisted thoracoscopic surgery; DFI, disease-free interval.

## Data Availability

The data presented in this study are available upon request from the corresponding author.
